# Polar Coding and Early SIC Decoding for Uplink Heterogeneous NOMA

**DOI:** 10.3390/e27111167

**Published:** 2025-11-18

**Authors:** Chu-Jung Wu, Chien-Ying Lin, Yu-Chih Huang

**Affiliations:** 1 Institute of Artificial Intelligence Innovation, National Yang Ming Chiao Tung University, Hsinchu 300093, Taiwan; gina.wu.ii11@nycu.edu.tw; 2 Institute of Communications Engineering, National Yang Ming Chiao Tung University, Hsinchu 300093, Taiwan; thisistt.c@nycu.edu.tw

**Keywords:** NOMA, early decoding, polar code

## Abstract

In modern communication systems, packets with different blocklengths often coexist, presenting new challenges for interference management and decoding. In scenarios where short-packet transmissions must meet strict latency and reliability requirements, conventional interference cancellation decoding strategies may be insufficient, especially when coexisting with long-packet services. This work proposes a novel interleaver design for polar codes that enables early decoding in successive interference cancellation (SIC) frameworks. To support this capability, a minimal yet essential modification to the interleaver used in the 5G New Radio (NR) polar coding scheme is introduced. This tailored interleaver facilitates the reliable recovery of short-packet signals before the complete decoding of coexisting long packets, substantially improving early decoding performance. Importantly, the proposed modification retains compatibility with the overall 5G NR polar code structure, ensuring practical implementability. Simulation results demonstrate that our approach yields significantly enhanced decoding accuracy in heterogeneous traffic scenarios representative of next-generation wireless systems.

## 1. Introduction

The 5G communication system and its successors are expected to support a wide range of services, such as enhanced mobile broadband (eMBB) and Ultra-Reliable Low-Latency Communication (URLLC) [1]. To enable the coexistence of heterogeneous services like URLLC and eMBB within the same network architecture, network slicing [2] is used to ensure service isolation and meet quality-of-service guarantees. However, it requires allocating orthogonal resources to each service. Such an approach will make it difficult to support a growing number of devices and services due to inefficient resource usage. This challenge has led to increasing attention from both academia and industry on developing more efficient coexistence mechanisms.

Research has been conducted to explore efficient coexistence mechanisms for heterogeneous services [3], aiming to enhance efficiency and user fairness. One possible solution is the use of non-orthogonal multiple access (NOMA) with superposition coding and successive interference cancellation (SIC) decoding to enable the coexistence of heterogeneous services. However, a key challenge in heterogeneous scenarios is that URLLC transmissions may not be able to utilize SIC due to their strict latency constraints. In order to perform SIC, the entire transmission frame, longer than the URLLC packet length, must be received, introducing significant latency for URLLC services. To address this issue, ref. [3] introduced heterogeneous NOMA by always forcing the receiver to decode URLLC messages first. As a result, the achievable rates of URLLC and other heterogeneous users can be significantly reduced in some cases, potentially falling below that of orthogonal multiple access (OMA). In addition, if the interference is not properly managed, it can significantly degrade the reliability of URLLC services, preventing them from meeting their stringent reliability requirements.

To reduce the reception latency of SIC, refs. [4,5] leveraged the concept of early decoding [6] for the downlink broadcast channel. These studies demonstrate that, under certain conditions, it is theoretically possible for a URLLC user to decode and subtract the eMBB messages after receiving only a fraction of the superimposed coded symbols. However, the proposed schemes therein are based on Gaussian signaling and random codebooks, which are not directly applicable to practical communication systems that employ binary coded modulations. In contrast, refs. [7,8] proposed a heterogeneous coded modulation scheme and employed treating interference as noise (TIN) decoding at the receiver. To practically approach the performance under perfect SIC, the scheme adopts single-user binary codes while carefully designing both constellations and power allocation. Specifically, sub-blocks of each codeword are mapped to tailored constellations, and the constellations corresponding to different sub-blocks may vary. However, these schemes still incur performance loss compared to the idealized scenario assuming perfect SIC. To enable interference cancellation with low-latency constraint, this paper aims to design of new coding schemes tailored for early decoding-based SIC in uplink multiple access channels.

Polar codes were recently proposed by Arıkan [9]. It is the first class of coding schemes with deterministic structures that are proven to achieve the capacity of symmetric binary-input discrete memoryless channels (B-DMCs) under low-complexity encoding and decoding. After its invention, polar codes have attracted significant attention in both academia and industry, leading to extensive research on their theoretical properties [10,11,12], improved constructions [13,14,15,16,17], decoding algorithms [18,19], and practical enhancements [20,21]. Notably, polar codes have been adopted as the channel coding scheme for control channels in the 5G New Radio (NR) standard due to their excellent error-correcting performance and structured design. In particular, various improvements such as successive cancellation list (SCL) decoding [18], CRC-aided polar codes [13], and interleaving techniques have been developed to boost their finite-length performance and adapt them for practical use cases.

In light of the high success of polar codes, this paper presents a novel approach to polar coding tailored for early-decoding-based interference cancellation decoding in the uplink multiple access channel. The key contributions include the design of a specialized interleaver based on the newly introduced notion of the *degree of mixture*, which optimizes the ordering of coded bits to enhance decoding accuracy for URLLC signals while facilitating early decoding, thus addressing strict latency constraints. Simulation results demonstrate that, with only a minimal modification to the 5G NR polar codes, the proposed method significantly outperforms the original 5G NR polar codes specified in 3GPP standards, particularly at mid- to high code rates, thereby enhancing URLLC performance in the presence of interference from eMBB services. This research opens pathways for further exploration into optimizing communication strategies within heterogeneous service environments, emphasizing the importance of effective decoding mechanisms for future wireless communication systems.

### Notation

In this paper, scalars are denoted by lowercase letters (e.g., *x*), vectors by bold lowercase letters (e.g., x), matrices by bold uppercase letters (e.g., X), and sets by calligraphic letters (e.g., X).

## 2. System Model and Polar Codes Preliminaries

In this section, we first introduce the system model—heterogeneous multiple access uplink with one eMBB user and one URLLC user. We characterize the signal received by the base station, clarify the error rate to evaluate the performance of early decoding. Next, we review the construction method of polar codes and the decoding method. Last, we briefly introduce the polar code standard in 5G NR, focusing on the sub-block interleaver and channel interleaver, which are critical to the main idea of designing our new interleaver.

### 2.1. Problem Formulation: Coding for Heterogeneous Multiple Access

**Figure 1:**
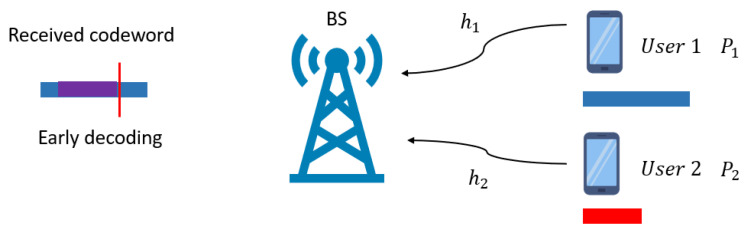
Two-user uplink model, where user 1 sends a longer codeword (blue block) and user 2 sends a shorter codeword (red block). The base station receives the superimposed signal, where the overlapped part is highlighted in purple.

The system considered in this study is a two-user uplink scenario as shown in Figure 1. We let $N_{k}$ represent the length of the symbol block for user *k*, where k∈{1,2}. Without loss of generality, we assume that user 2 has a more stringent latency requirement than user 1, and therefore $N_{1}>N_{2}$. Accordingly, throughout the paper, users 1 and 2 are referred to as eMBB and URLLC users, respectively. We denote by $x_{1}$ and $x_{2}$ the signals transmitted by users 1 and 2, respectively. Each user’s transmitted signal is subject to a power constraint given by

(1) $$\frac{1}{N_{k}}\sum_{i=1}^{N_{k}}{\left|x_{k}\left[i\right]\right|}^{2}\leq P_{k},\;k\in \left\{1,2\right\}.$$

The base station receives a superimposed signal y:

(2) $$y\left[i\right]=\left\{\begin{array}{cc} h_{1}x_{1}\left[i\right]+z\left[i\right], & i=1,\ldots ,n_{s},\\ h_{1}x_{1}\left[i\right]+h_{2}x_{2}\left[i-n_{s}\right]+z\left[i\right], & i=n_{s}+1,\ldots ,n_{s}+N_{2},\\ h_{1}x_{1}\left[i\right]+z\left[i\right], & i=n_{s}+N_{2}+1,\ldots ,N_{1}, \end{array}\right.$$

where $h_{k}$ denotes user *k*’s channel, z[i] represents the Gaussian noise of variance $\sigma^{2}$ at the *i*-th time instant, $n_{s}+1$ is the start time of the URLLC transmission with $n_{s}+N_{2}\leq N_{1}$.

To ensure the strict latency constraint is met, for the URLLC signal, decoding takes place immediately after the reception of $y\left[1\right],\ldots ,y\left[n_{s}+N_{2}\right]$ symbols. At this point, it is obvious that the eMBB signal of length $N_{1}$ may not be fully received, preventing traditional homogeneous NOMA schemes from performing SIC successfully. In this work, we consider the process of starting decoding significantly earlier than the complete reception of a codeword, which is referred to as *early decoding*. As shown in Figure 1, the base station receives the superposition of the two users’ signals and attempts to decode the eMBB signal using only the first $n_{s}+N_{2}$ received symbols. Meanwhile, for decoding the eMBB signal, the remaining $N_{1}-N_{2}-n_{s}$ symbols with indices $n_{s}+N_{2}+1,\ldots ,N_{1}$ continue to arrive at the base station, and user 1’s codeword can then be decoded by canceling the previously decoded ${\hat{x}}_{2}$ from y, similar to the decoding procedure in homogeneous NOMA.

Let ${\tilde{x}}_{1}$ denote the early-decoded version of $x_{1}$, which has length $N_{1}$. It is obtained using only the first $n_{s}+N_{2}$ received symbols for decoding and has a BLER of

(3) $${\tilde{p}}_{e,1}=P\left({\tilde{x}}_{1}\neq x_{1}\right).$$

Here, ${\tilde{p}}_{e,1}$ reflects the error rate of the first stage of SIC decoding, which serves only to facilitate user 2’s decoding, and is not the final error rate of user 1’s transmission. The base station then cancels ${\tilde{x}}_{1}$ from the superimposed signal, leaving only user 2’s signal, which is decoded to form the estimate ${\hat{x}}_{2}$ with BLER:

(4) $$p_{e,2}=P\left({\hat{x}}_{2}\neq x_{2}\right).$$

It is important to note that ${\tilde{x}}_{1}$ is used solely for early SIC and does not serve as the final decision for user 1. Once the entire block is received, the base station re-decodes user 1’s signal with the full set of received symbols to obtain the final estimate $\hat{x}1$, yielding a BLER:

(5) $$p_{e,1}=P\left({\hat{x}}_{1}\neq x_{1}\right).$$

In traditional homogeneous NOMA systems, where $N_{1}=N_{2}$, channel ordering ensures a low early-decoding error probability ${\tilde{p}}_{e,1}$ by relying on codes that inherently achieve a small $p_{e,1}$. In contrast, in heterogeneous NOMA with $N_{1}>N_{2}$, channel ordering alone is insufficient, thereby motivating the need for new code designs. The central idea is to construct codes that can be reliably decoded with the minimum number of received symbols. To this end, this paper develops a new class of codes that support early decoding. That is, codes that achieve low ${\tilde{p}}_{e,1}$ when decoded prematurely at length $n_{s}+N_{2}$, while maintaining $p_{e,1}$ performance comparable to existing codes when decoded in full at length $N_{1}$.

To realize this objective, we design a new polar coding scheme that enables early decoding. In the 5G NR polar coding procedure, there is a step called rate matching, which adjusts the codeword length to satisfy channel constraints. This is typically achieved via puncturing and shortening, where certain bits are removed from the original codeword. Even after such modifications, the codeword remains decodable despite some bits being missing. This observation naturally raises the question of which specific bits should be prioritized for removal to optimize early-decoding performance—a question that lies at the heart of our proposed design.

### 2.2. Polar Codes

**Figure 2:**
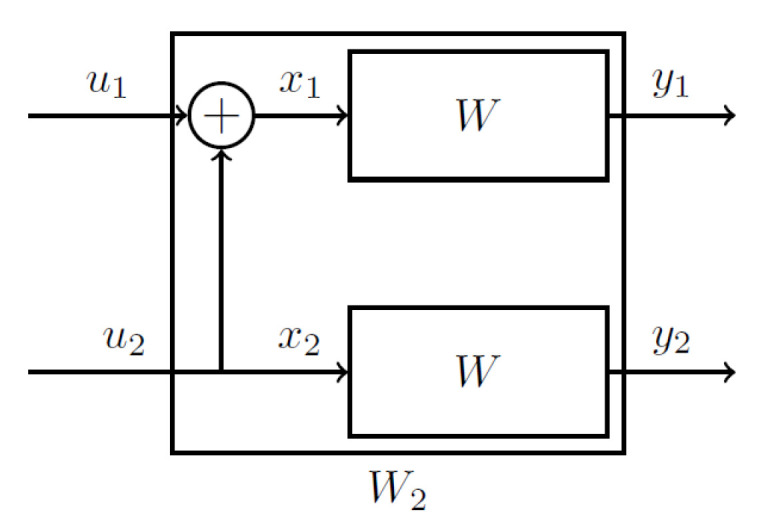
Basic transformation adopted in polar codes.

In this section, we review some basic concepts of polar codes. Consider a B-DMC W:X→Y, where X and Y denote the input and output alphabets, respectively. The channel transition probability is defined as W(y∣x) for x∈X,y∈Y. Consider the basic transformation illustrated in Figure 2 and given by

(6) $$G_{2}=F=\left[\begin{array}{cc} 1 & 0\\ 1 & 1 \end{array}\right]$$

The generator matrix of a length $N=2^{n}$ polar code is constructed as $G_{N}=F⊗G_{N/2}$ where ⊗ denotes the Kronecker product. For example, for N=4, we have

(7) $$G_{4}=\left[\begin{array}{cccc} 1 & 0 & 0 & 0\\ 1 & 1 & 0 & 0\\ 1 & 0 & 1 & 0\\ 1 & 1 & 1 & 1 \end{array}\right].$$

As shown in [9], encoding with $G_{N}$, combined with successive cancellation (SC) decoding, leads to channel polarization, wherein each bit channel becomes either nearly perfect or highly noisy. The central idea behind polar codes is to *freeze* the highly noisy channels and transmit uncoded information bits through the nearly perfect ones.

Specifically, taking advantage of channel polarization, we select the noiseless channels for transmission to ensure the reliability of the information. After applying polarization on $N=2^{n}$ independent instances of *W*, we obtain *N* binary-input channels $W_{N}^{\left(i\right)}$ for i=0,1,…,N−1. Information bits are assigned to the channels in the information set A, representing the reliable channels. The complement set $A^{c}$ is the frozen set, used for noisy channels, where frozen bits $u_{A^{c}}$ (known to both the transmitter and receiver) are transmitted. The code rate is defined as R=|A|/N. The construction of polar codes, i.e., identifying A and $A^{c}$ can be done offline and the complexity is amortized.

SC decoding is one of the common methods for decoding polar codes. The SC decoder decodes bits sequentially, making decisions based on previously decoded bits and the current channel observation. It has a complexity of O(NlogN). However, it performs poorly in practical blocklength regimes because early wrong decisions cannot be corrected later. The SCL decoding  [18] addresses this issue by keeping multiple decoding paths in parallel. Instead of following a single path, it considers up to *L* most probable decoding paths. The decoded codeword is then chosen as the path with the highest likelihood among the final *L* candidates. When combined with a Cyclic Redundancy Check (CRC), CRC-aided SCL (CA-SCL) decoding [13] performs better than plain SCL, as the CRC is used to validate the final candidates.

### 2.3. Polar Codes in 5G NR

In 5G NR, polar codes are implemented for control channels, with distinct coding procedures for uplink and downlink as specified in 3GPP TS 38.212 [22]. To employ polar codes described in Section 2.2 for commercial systems such as 5G, many additional challenges need to be addressed. Among them, the most crucial step in 5G polar encoding is the rate matching. Rate matching involves three stages: sub-block interleaving, bit selection, and channel interleaving. According to 3GPP [22], bit selection is not required in the uplink.

The sub-block interleaver reorders the codeword after polar encoding based on channel reliability. It divides the codeword into 32 sub-blocks and rearranges them using a pre-defined pattern that roughly follows subchannel reliability. Though not exact, it is computationally efficient and adaptable to varying code lengths.

**Figure 3:**
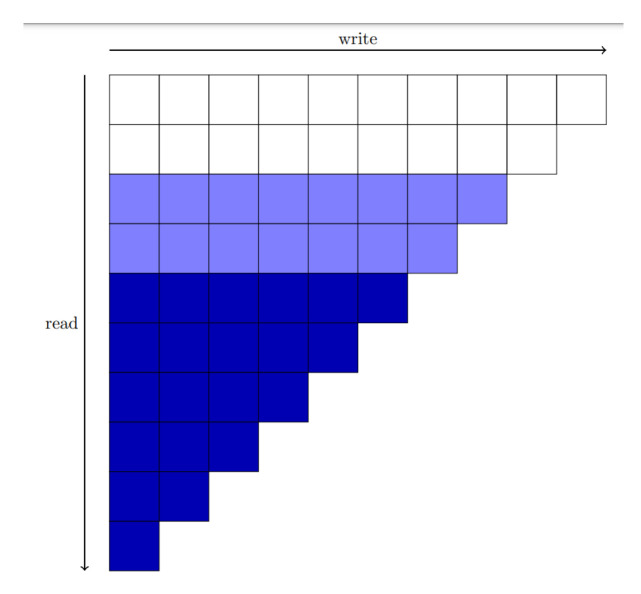
Channel interleaver. The data is filled row-wise from top to bottom and read column-wise from left to right. The portion filled later (dark blue blocks) will constitute the majority of the earlier part of the received sequence.

Channel interleaving is specific to uplink channels. To mitigate interference between adjacent coded bits, the interleaver disperses them, which is especially important for higher-order modulations. The channel interleaver used in 3GPP 5G NR is shown in Figure 3, where bits are filled in row-wise and read out column-wise.

In early decoding scenarios, 5G NR polar codes may encounter several challenges. Firstly, these polar codes are designed for complete codeword decoding, relying on the full reception of the codeword to ensure high accuracy. Consequently, when only a partial codeword is received, the reliability of the decoding process may decrease significantly. Secondly, the channel reliability sorting employed in the design of polar codes is based on the entire codeword length; this can lead to suboptimal prioritization of information bits during early decoding, adversely affecting decoding performance. Additionally, the complexity of the 5G NR system and its stringent low-latency requirements may make successful decoding increasingly difficult when partial information is unavailable, raising the likelihood of early decoding failures.

## 3. Proposed Method

In this section, we propose a novel interleaver design for polar codes that facilitates early decoding based on the newly introduced notion of the degree of mixture and its corresponding partitioning scheme. The objective is to improve decoding performance in heterogeneous multiple-access scenarios, particularly in situations where only a partial portion of the codeword is available at the receiver.

### 3.1. Degree of Mixture, Subchannel Partitions, and Group Variances

We now introduce the definitions that are essential to the proposed interleaver design. In the standard 5G NR polar code design, the sub-block interleaver is optimized for full-length decoding and is not tailored to early-decoding scenarios, where decoding must commence before the entire codeword is received. To overcome this limitation, we propose an alternative ordering scheme that leverages the structure of Pascal’s triangle. The key idea is to prioritize sub-channels with a moderate degree of mixture, thereby ensuring that the symbol distribution in the initial portion of the received codeword closely resembles that of a full-length polar code.

**Definition 1.**  *For j∈{0,1,…,N−1}, the symbol $x_{j}$ corresponding to subchannel j of a polar code is said to be the mixture of $u_{\ell_{1}},\ldots ,u_{\ell_{d}}$ for d distinct $\ell_{1},\ldots ,\ell_{d}\in \left\{0,\ldots ,N-1\right\}$ if*

(8) $$x_{j}=\overset{d}{\underset{i=1}{⨁}}u_{\ell_{i}},$$

*where d is called the degree of mixture. On the other hand, these $u_{\ell_{1}},\ldots ,u_{\ell_{d}}$ are said to be components of $x_{j}$. By the structure of polar code, d can only be $2^{i}$ for 0≤i≤n.*

We observe that the degree of mixture for each subchannel index $x_{i}$ can be readily determined from the binary representation of *i*. Specifically, in the *n*-bit binary representation of *i*, each zero bit causes the degree of mixture to double. For instance, $x_{5}$ corresponds to the binary sequence 0101 and is expressed as

(9) $$x_{5}=u_{5}⊕u_{7}⊕u_{13}⊕u_{15},$$

which yields a degree of mixture equal to 4.

**Definition 2.**  *For a $N=2^{n}$ polar code,*

(10) $${\left\{G_{k}\right\}}_{k=0}^{m}=\left\{G_{0},G_{1},\ldots ,G_{m}\right\},\;0\leq m\leq N-1,$$

*are said to form a partition of subchannels ${\left\{x_{j}\right\}}_{j=0}^{N-1}$ if the assignment of subchannels to groups satisfies the following conditions:*

1. ***Unique Membership:*** *Each subchannel $x_{j}$ is assigned to exactly one group $G_{k}$. Formally,*
(11) $$\forall j\in \left\{0,\ldots ,N-1\right\},\;\exists !\;k\in \left\{0,\ldots ,m\right\}\;\mathrm{such}\;\mathrm{that}\;x_{j}\in G_{k}.$$
2. * **Partition Property:** The groups $G_{k}$ form a partition of $\left\{x_{0},\ldots ,x_{N-1}\right\}$, i.e.,*
(12) $$\overset{m}{\underset{k=0}{⋃}}G_{k}=\left\{x_{0},x_{1},\ldots ,x_{N-1}\right\},\;G_{k}\cap G_{k^{\prime }}=\emptyset \;\mathrm{for}\;k\neq k^{\prime }.$$

**Definition 3.**  *For each group $G_{k}$, let $A_{k}$ consists of the number of times $u_{i}$ is a component of $x_{j}$ for all $x_{j}\in G_{k}$. We define $\mathrm{var}(G_{k})$ the variance of the group $G_{k}$ as the variance of a random variable uniformly distributed over the elements of $A_{k}$.*

**Figure 4:**
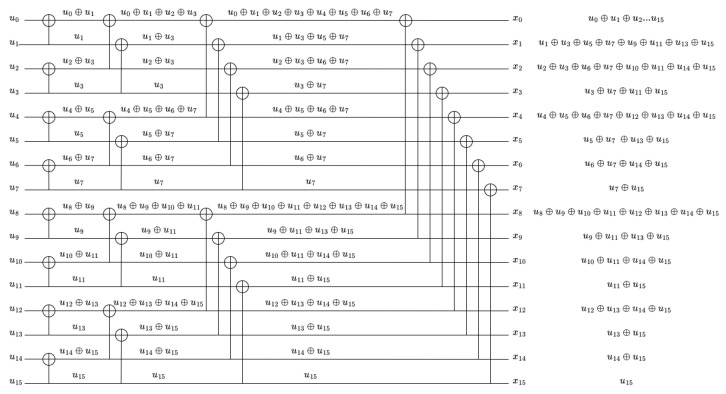
For N=16, the number of information sent in polarized channels is in Pascal sequence behavior.

An example is given in Figure 4, where we consider a polar code of N=16. Let $G_{k}=\left\{x_{15},x_{14},x_{13}\right\}$, corresponding to the mixtures of components of $\left\{u_{15},u_{15}⊕u_{14},u_{15}⊕u_{13}\right\}$, respectively. It is apparent that $u_{15}$ appears three times, while the others appear once. Thus, we have $A_{k}=\left\{3,1,1\right\}$ and the variance of $G_{k}$ is $\mathrm{var}(G_{k})=0.88$.

If all subchannels are in a single group, that is, the complete polar code, we can observe that the variance is large. This is because the structure of polar codes causes the input components to be mixed in varying numbers of subchannels, which can differ significantly. In general, input components with larger indices are mixed in more subchannels, while those with smaller indices are mixed in fewer subchannels. The huge difference between the number of components is what makes the variance large. Take N=16 for example, we have $A_{0}=\left\{1,2,2,4,2,4,4,8,2,4,4,8,4,8,8,16\right\}$, the variance of $G_{0}=\left\{x_{0}^{15}\right\}$ is 13.43.

### 3.2. Proposed Interleaver for Early Decoding

Motivated by the above observation, our proposed method for early decoding is to make the received component distribution pattern behave more closely to the complete polar code in the early stage of receiving. Note that with early decoding, the component distribution of the early received portion will never exactly match that of the complete polar code. However, we can make the ratio between components resemble that of the complete polar code as closely as possible.

Our proposed method classifies the subchannel indices of a polar code with kernel size $N=2^{n}$ into n+1 groups according to their degrees of mixture. Specifically, for $N=2^{n}$, let $G_{k}$ denote the set of subchannels whose degree of mixture equals $2^{k}$, where k∈{0,1,…,n}. The indices belonging to each group $G_{k}$, for 0≤k≤n, can be expressed as

(13) $$G_{k}=\{x_{i}|\mathrm{the}\;\mathrm{binary}\;\mathrm{representation}\;\mathrm{of}\;i\;\mathrm{contains}\;k\;\mathrm{zeros}\},$$

The cardinality of each group $G_{k}$ can be directly computed as

(14) P(n,k)=nk=n!k!(n−k)!, 0≤k≤n,

indicating that there are P(n,k) subchannels sharing the *same* degree of mixture $2^{k}$.

To assess the relative importance of each group for early decoding, we compute the variance of the component counts within each group $G_{k}$. The mixture components of a subchannel can be identified by tracing back the recursive structure of the polar code. Algorithm 1 presents the procedure for calculating the group variance based on the allocation of subchannels according to their degrees of mixture. Specifically, the arrays seq and previous_seq are both arrays of sequences, where each sequence records the component counts for subchannels in a given group $G_{k}$. For a polar code of length $N=2^{n}$, there are n+1 groups, so the array seq will eventually contain n+1 sequences. Interestingly, as the structure of the polar code expands, the component counts of each group exhibit a triangular relationship reminiscent of Pascal’s triangle. When the kernel size doubles, the sequences of each group are updated by performing element-wise addition of the two sequences above, appending the upper-left sequence, and finally sorting the resulting sequence in descending order.
**Algorithm 1** $\mathrm{Calculate}\;\mathrm{the}\;\mathrm{Group}\;\mathrm{variance}\;\mathrm{with}\;\mathrm{kernel}\;size\;(N=2^{n})$.  1:Initialize seq[0…n]←[null]  2:Initialize previous_seq[0…n]←[null]  3:previous_seq[0]←[1]  4:**for** i←0 to *n* **do**  5:      **for** j←0 to *i* **do**  6:            seq[j]← element-wise sum of previous_seq[j−1] and previous_seq[j]  7:            Append previous_seq[j−1] to the end of seq[j]  8:            Sort seq[j] in descending order  9:      **end for**10:      previous_seq←seq11:**end for**12:**for** i←0 to *n* **do**13:      var_array[i]←Variance(seq[i])14:**end for**15:**return** var_array

According to the grouping strategy, groups with larger sizes, corresponding to more moderate degrees of mixture, exhibit higher variances. Among groups with the same size, the one with a smaller degree of mixture shows a higher variance. For any given *N*, the ordering of group variances follows a fixed pattern: it begins from the middle group in Pascal’s triangle and then expands symmetrically toward both sides, one group at a time.

To emulate the behavior of the complete polar code during the early stage of reception, the group with the highest variance is regarded as the most important. The importance of all groups is therefore determined by their variances, arranged in descending order. To ensure that the more significant groups are prioritized earlier in the received sequence, high-variance groups are placed toward the lower part of the 5G NR triangular channel interleaver, effectively filling the interleaver from the lowest-variance group to the highest-variance group. Within each group, subchannels with larger indices are considered more important and are thus positioned later within that group in the interleaver.

During the early reception stage, symbols from high-variance groups dominate the received portion, while symbols from lower-variance groups are mixed in. Consequently, the resulting component distribution exhibits higher variance than that of any single group alone, producing a distribution pattern that more closely approximates that of the complete polar code.

Algorithm 2 summarizes the procedure for generating the proposed interleaver sequence for a given *N*. Since the degrees of mixture, group compositions, and variance-based ordering of groups are fixed for each *N*, it is unnecessary to recompute the group variances during construction. The array level stores the possible degrees of mixture for each subchannel, namely N,N/2,…,1. The array polar records the information content at each stage of polarization, while info_sum stores its accumulated result for the given kernel size *N*. The array pascal_order specifies the transmission order of groups according to their variances, aligned with the degrees of mixture in level. The group-wise filling process, described in lines 26–38, follows the order discussed above. Finally, the interleaver is constructed by selecting the subchannels corresponding to the degrees of mixture in level, following the ordering defined by pascal_order.
**Algorithm 2** $\mathrm{Proposed}\;\mathrm{interleaver}\;\mathrm{for}\;\mathrm{kernel}\;\mathrm{size}\;(N=2^{n})$.  1:Initialize variables:num←n+1sum←N  2:Declare arrays: pascal_order[num], info_sum[N], level[num], interleaver[N]  3:**for** i←num−1 to 0 **do**  4:      level[i]←sum  5:      sum←sum/2  6:**end for**  7:**for** i←0 to N−1 **do**  8:      info_sum[i]←pow(2,n−conut_ones_in_binary(i))  9:**end for**10://Set the order for each group by variance11:**if** num is even **then**12:      **for** i←0 to num/2−1 **do**13:            pascal_order[i]←2(i+1)14:      **end for**15:      **for** i←0 to num/2−1 **do**16:            pascal_order[i+num/2]←num−1−2i17:      **end for**18:**else**19:      **for** i←0 to (num−1)/2−1 **do**20:            pascal_order[i]←2(i+1)21:      **end for**22:      **for** i←0 to (num+1)/2−1 **do**23:            pascal_order[i+(num−1)/2]←num−2i24:      **end for**25:**end if**26://Make interleaver27:j←028:**for** k←1 to num **do**29:      Find *m* such that pascal_order[m]=k30:      now←m31:      **for** i←0 to N−1 **do**32:            **if** info_sum[i]=level[now] **then**33:                  interleaver[j]←i34:                  j←j+135:            **end if**36:      **end for**37:**end for**38:**return** interleaver

This sequence is then passed through the 5G NR triangular channel interleaver, similar to the standard procedure. As shown in Figure 3, the triangular interleaver ensures that later filled bits are received earlier during transmission. By placing the more important subchannels at the bottom of the triangle, we ensure that these bits are prioritized during early reception.

Our proposed method is a universal design for polar codes with $N=2^{n}$. Due to the structure of polar codes, grouping by degrees of mixture is always applicable. By leveraging the transmission order of the triangle channel interleaver, we prioritize subchannels in high-variance groups, thereby improving decoding performance.

In what follows, we present a detailed example with n=4.

**Example 1.**  *For a polar code with block length $N=2^{4}=16$, as illustrated in Figure 4, the subchannels are categorized according to their degrees of mixture, namely 1, 2, 4, 8, and *16*. Each group respectively contains 1, 4, 6, 4, and *1* subchannels, which aligns with the fifth row of Pascal’s triangle. Our grouping method is based on the degree of mixture, where $G_{k}$ denotes the set of subchannels whose degree of mixture is $2^{k}$. The group partitions are given by*

(15) $$\begin{array}{cc} G_{0} & =\{x_{15}\}, \end{array}$$

(16) $$\begin{array}{cc} G_{1} & =\{x_{7},x_{11},x_{13},x_{14}\}, \end{array}$$

(17) $$\begin{array}{cc} G_{2} & =\{x_{3},x_{5},x_{6},x_{9},x_{10},x_{12}\}, \end{array}$$

(18) $$\begin{array}{cc} G_{3} & =\{x_{1},x_{2},x_{4},x_{8}\}, \end{array}$$

(19) $$\begin{array}{cc} G_{4} & =\{x_{0}\}. \end{array}$$

*Based on these groupings, we compute the group variances to quantify their relative importance for early decoding. For example, consider $G_{1}=\left\{x_{7},x_{11},x_{13},x_{14}\right\}$, whose subchannels represent mixtures of the components:*

$$\{u_{7}⊕u_{15},\;u_{11}⊕u_{15},\;u_{13}⊕u_{15},\;u_{14}⊕u_{15}\}.$$

*The corresponding component count vector is given by $A_{1}=\left(4,1,1,1,1\right)$, which yields a group variance of 1.44. According to Algorithm 1, the variances of the individual groups are computed as*

(20) $$\begin{array}{cc} \mathrm{var}(G_{0}) & =0, \end{array}$$

(21) $$\begin{array}{cc} \mathrm{var}(G_{1}) & =1.44, \end{array}$$

(22) $$\begin{array}{cc} \mathrm{var}(G_{2}) & =2.33, \end{array}$$

(23) $$\begin{array}{cc} \mathrm{var}(G_{3}) & =0.78, \end{array}$$

(24) $$\begin{array}{cc} \mathrm{var}(G_{4}) & =0. \end{array}$$

*Consequently, the groups can be ranked in descending order of their variances as*

(25) $$G_{2}>G_{1}>G_{3}>G_{0}=G_{4}.$$

*Accordingly, the transmission order of the subchannel groups is*

(26) $$[G_{4},\;G_{0},\;G_{3},\;G_{1},\;G_{2}],$$

*which translates explicitly to the subchannel sequence*

(27) $$[x_{0},x_{15},x_{1},x_{2},x_{4},x_{8},x_{7},x_{11},x_{13},x_{14},x_{3},x_{5},x_{6},x_{9},x_{10},x_{12}].$$

*When the proposed Algorithm 2 is applied with N=16, the array level is [1,2,4,8,16], info_sum is [16,8,8,4,8,4,4,2,8,4,4,2,4,2,2,1], and pascal_order is [2,4,5,3,1]. The resulting interleaver is then mapped into the triangular channel interleaver structure of 5G NR following this order.*

## 4. Simulation

In this section, we present simulation results to highlight the effectiveness of the proposed approach. Three methods are considered: the 3GPP 5G NR polar code procedure ([22], Section 2.3), our proposed method, and a benchmark method that arranges subchannel groups from the highest to the lowest degree of mixture. The primary difference among the three lies in how the code bits are arranged during the sub-block interleaving step in the 3GPP procedure. It is important to note that bit selection is not considered in this simulation. Moreover, no bits are repeated, shortened, or punctured.

For all simulations, we consider 11-bit CRC and SCL decoding with list size 4. Here, we define the receive rate ρ as the ratio of the partially received codeword (when the codeword for user 2 is received) to the full-length codeword. A receive rate of 0.5 means that only the first half of the codeword is received for early decoding. For the portion of the codeword not received, it is treated as being punctured, i.e., having LLR (Log-Likelihood Ratio) of 0, implying equal probability for the bit to be 0 or 1. For modulation, we present results for BPSK with the benchmark method, as well as for 16-QAM, 64-QAM, and 256-QAM, using both the 5G NR and our proposed method.

### 4.1. Benchmark

The benchmark is constructed based on the grouping allocation according to the degree of mixture, with the group order arranged in reverse, from index *n* to 0. This design ensures that subchannels with lower degrees of mixture dominate the earlier portion of the received sequence. The benchmark serves to underscore the superiority of the proposed method, which instead prioritizes groups with moderate degrees of mixture at the beginning.

Take N=16 for example, the transmission order of the benchmark for subchannel groups is given by $\left[G_{4},G_{3},G_{2},G_{1},G_{0}\right]$, and the full ordering is

(28) $$[x_{0},x_{1},x_{2},x_{4},x_{8},x_{3},x_{5},x_{6},x_{9},x_{10},x_{12},x_{7},x_{11},x_{13},x_{14},x_{15}].$$

### 4.2. Simulation for Single-User Early Decoding

For the single-user simulation, we consider one transmitter and one receiver, with only noise present and no other interference. As mentioned in Section 2.1, we evaluate the decoding performance in terms of error probability, which in the simulation is represented by BLER. Let x denote the signal of power *P* sent by the transmitter. The BLER is defined as ${\tilde{p}}_{e}=P\left(\tilde{x}\neq x\right)$, where $\tilde{x}$ is the decoded signal when x is not fully recovered. We simulate ${\tilde{p}}_{e}$ vs. $SNR=P/\sigma^{2}$.

**Figure 5:**
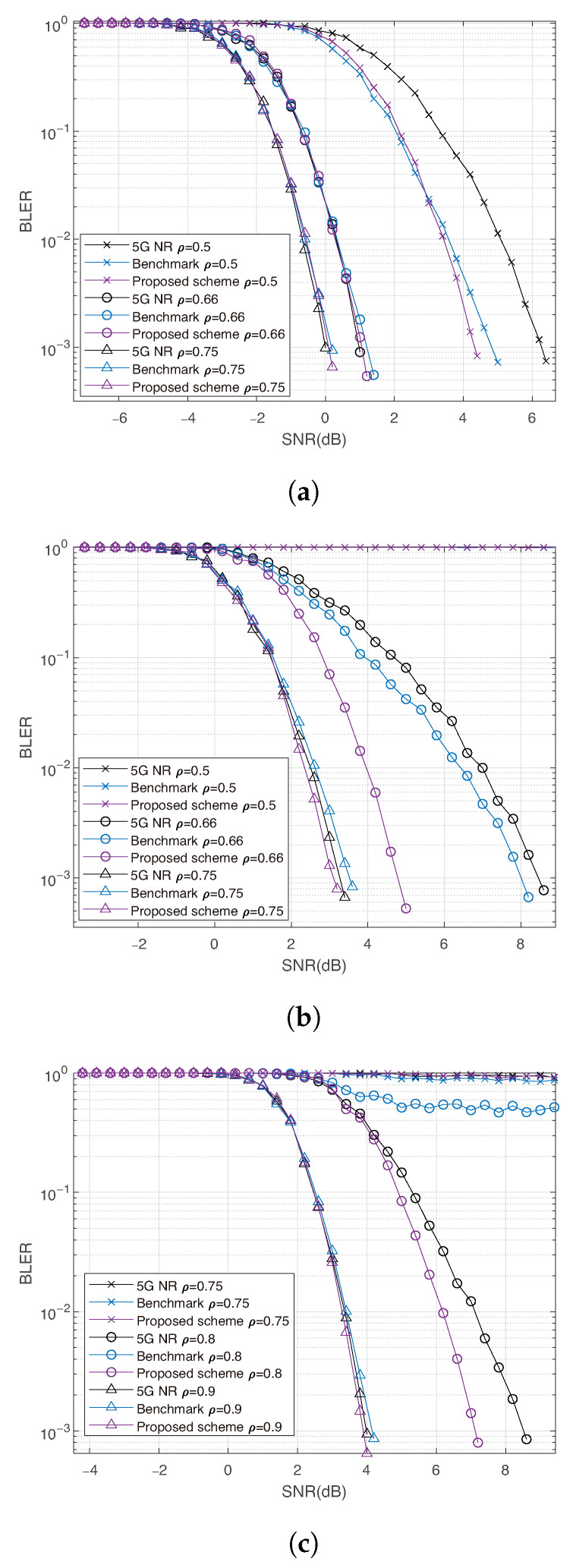
Single user simulation results using BPSK with N=256. (**a**) Comparison between the proposed method and benchmark at rate R=0.3. (**b**) Comparison between the proposed method and benchmark, and code rate R=0.5. (**c**) Comparison between the proposed method and benchmark at rate R=0.7.

Figure 5 shows the simulation results of BPSK for single-user early decoding. When the code rate is R=0.3, our method successfully decodes for ρ=0.5, 0.66, and 0.75, achieving a lower BLER than both 5G NR and the benchmark. For R=0.5, similar performance is observed for ρ=0.66 and 0.75. However, when ρ=0.5, none of the schemes can successfully decode due to insufficient received information. For R=0.7, all schemes fail when ρ=0.75. Overall, our method outperforms the benchmark in nearly all cases and surpasses 5G NR in most mid- and high-code-rate scenarios, particularly for longer blocklengths. However, it performs worse at low rates because, in such settings, most bits are frozen, which reduces the effectiveness of prioritizing moderate degree of mixture subchannels and thereby limits the gains from early decoding.

**Figure 6:**
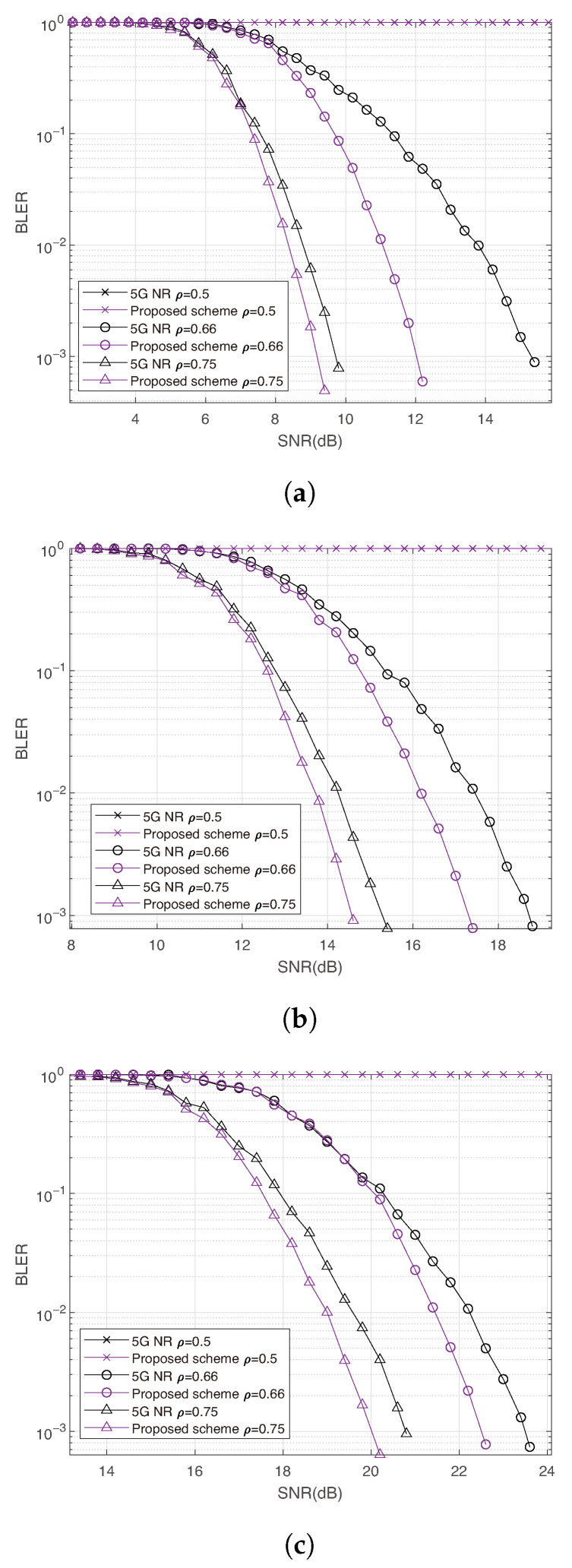
Single user simulation results using high order modulation with N=256, code rate R=0.5. (**a**) Comparison between the proposed method and 5G NR using 16-QAM. (**b**) Comparison between the proposed method and 5G NR using 64-QAM. (**c**) Comparison between the proposed method and 5G NR using 256-QAM.

Figure 6 shows the simulation result of 16-QAM, 64-QAM and 256-QAM for single-user early decoding, the results are similar to BPSK cases.

### 4.3. Simulation for Heterogeneous Multiple Access

Having examined the single-user case, we now consider the case in Section 2.1, where two users, i.e., eMBB and URLLC users, transmit coded symbols with different blocklengths to the base station. Once the URLLC codeword is fully received, early decoding is performed for the eMBB signal. Then, SIC is used to remove the eMBB interference before decoding URLLC. Here, we assume that the two users transmit with the same power, i.e., $P_{1}=P_{2}=P$, but experience different channel gains $h_{1}$ and $h_{2}$. Moreover, we let $R=|A_{1}|/N_{1}=\left|A_{2}\right|/N_{2}$, where $A_{1}$ and $A_{2}$ are the information sets for the polar codes adopted at the users 1 and 2, respectively.

During early decoding of the eMBB signal, some parts may overlap with the URLLC signal. For these overlapping segments, LLRs are calculated bit-by-bit according to the two users’ constellation points. Specifically, we set $n_{s}=\rho N_{1}-N_{2}$, where the base station first decodes user 1’s signal using only $\rho N_{1}$ received symbols, resulting in a block error rate (BLER) of ${\tilde{p}}_{e,1}=P\left({\tilde{x}}_{1}\neq x_{1}\right)$. Subsequently, SIC is employed to decode user 2’s signal, yielding $p_{e,2}=P\left({\hat{x}}_{2}\neq x_{2}\right)$. Since our primary focus is on early decoding capability, we only consider the performance in the first stage of the SIC process, characterized by ${\tilde{p}}_{e,1}=P\left({\tilde{x}}_{1}\neq x_{1}\right)$. We simulate ${\tilde{p}}_{e,1}$ (and $p_{e,2}$) vs. $SNR=P/\sigma^{2}$.

**Figure 7:**
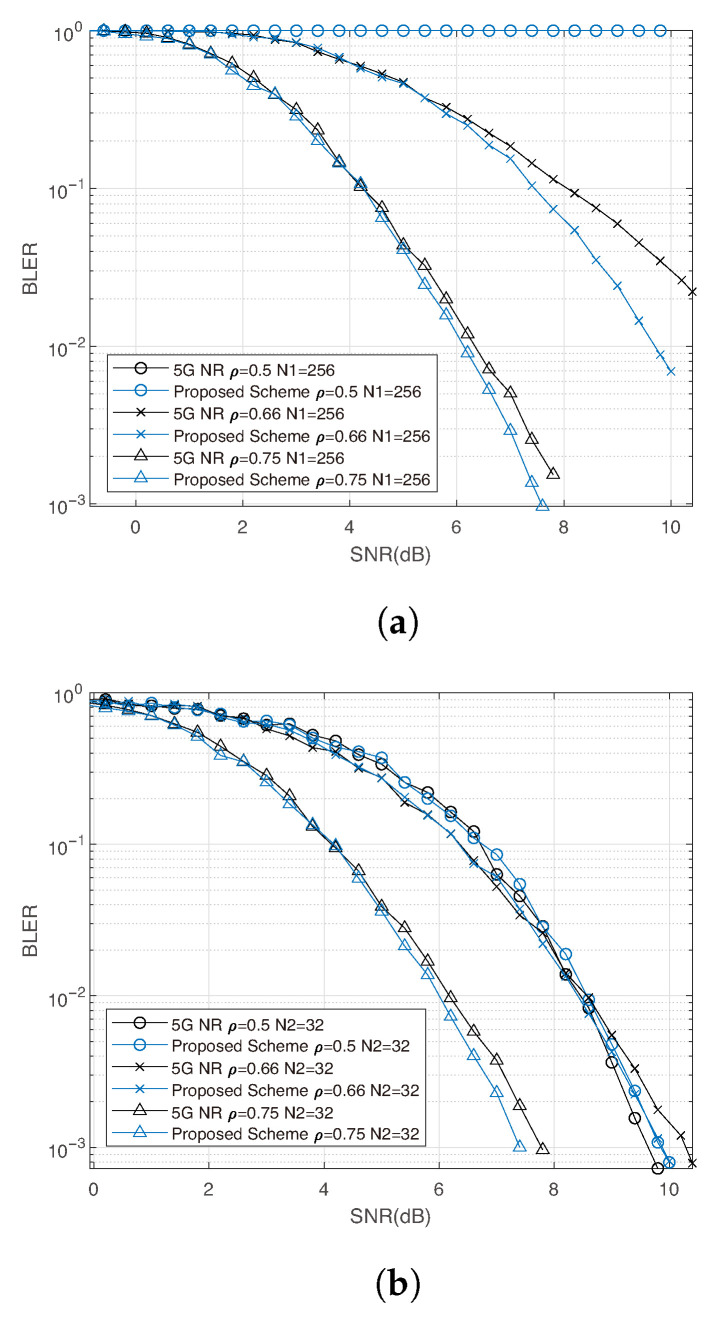
Uplink 2-user simulation results using BPSK, code rate R=0.5. (**a**) BLER of user 1 under early decoding and with $N_{1}=256$; (**b**) BLER of user 2 with $N_{2}=32$.

**Figure 8:**
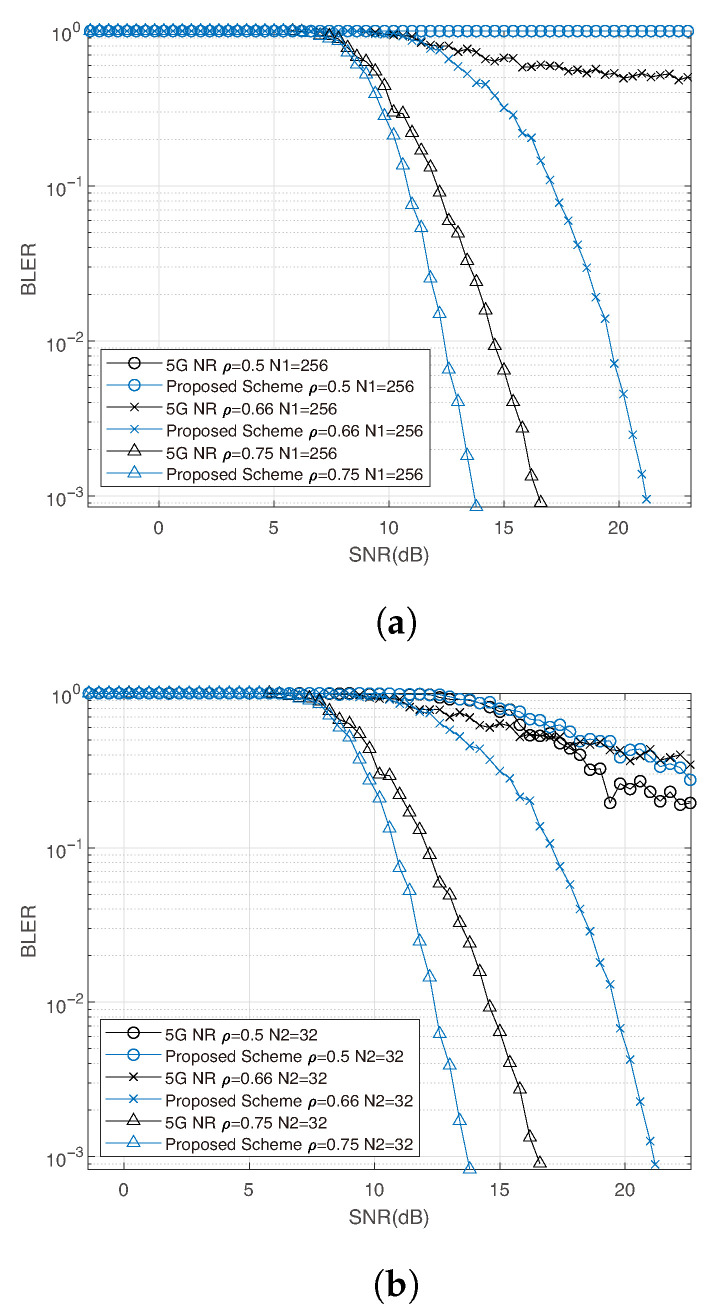
Uplink 2-user simulation results using 16-QAM, code rate R=0.5. (**a**) BLER of user 1 under early decoding and with $N_{1}=256$; (**b**) BLER of user 2 with $N_{2}=32$.

For the simulations in this section, user 1 and user 2 are assigned blocklengths of 256 and 32, respectively. The comparison is conducted for ρ∈{0.5, 0.66, 0.75}. Figure 7 presents the results obtained with BPSK modulation, while Figure 8 shows those with 16-QAM. For BPSK, we set $h_{1}=1$ and $h_{2}=1.5$; for 16-QAM, we set $h_{1}=1$ and $h_{2}=1.3$. The simulation results for this two-user scenario are consistent with the single-user case: the proposed interleaver achieves superior early decoding performance compared to the 5G NR baseline. At the same receive rate, our method enables more accurate eMBB decoding, thereby yielding a lower BLER for URLLC.

**Figure 9:**
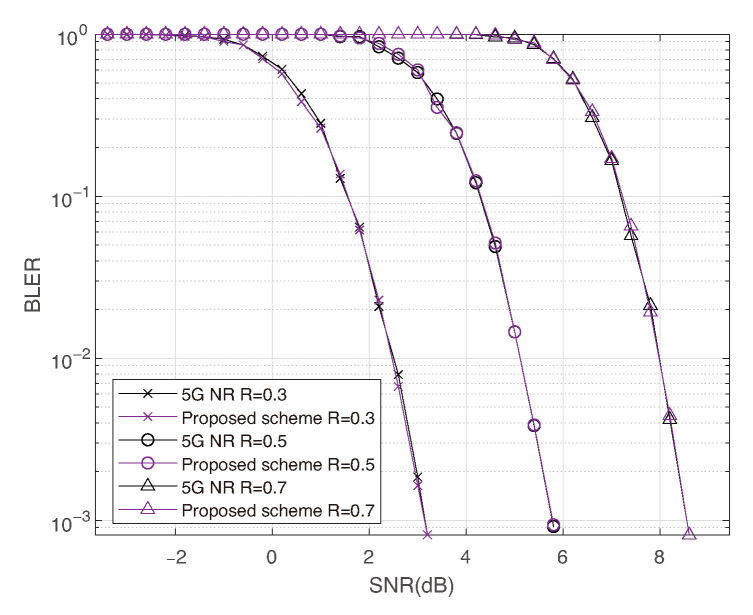
Comparison of 5G NR and proposed scheme under fully receive with 16-QAM, N=256, and code rate R=0.3,0.5,0.7.

Furthermore, we simulate the fully received cases (ρ=1) to examine whether the proposed design introduces additional channel interference between adjacent coded bits under high-order modulation. The results indicate that our design does not degrade decoding performance compared to the 5G NR standard. As shown in Figure 9, similar trends are observed across different code lengths, code rates, and modulation orders.

## 5. Conclusions

In this study, we investigate how heterogeneous services interfere with each other. To enable SIC decoding for URLLC in such systems, we perform early decoding, where the codeword is not fully received. We propose a new interleaver for polar codes, which facilitates early decoding in heterogeneous service systems and outperforms the existing 3GPP standard.

Our study has focused on the uplink scenario, but, for future work, the downlink scenario also warrants further investigation. Our proposed method could be further refined while maintaining the importance ordering. Additionally, rate matching was not considered in this study, so exploring how to integrate rate matching into our method represents another important avenue for future research.

## Data Availability

The datasets presented in this article are not readily available because the data are part of an ongoing study.
